# An Improved Transformation System for Cowpea (*Vigna unguiculata* L. Walp) via Sonication and a Kanamycin-Geneticin Selection Regime

**DOI:** 10.3389/fpls.2019.00219

**Published:** 2019-02-26

**Authors:** Bosibori Bett, Stephanie Gollasch, Andy Moore, Robert Harding, Thomas J. V. Higgins

**Affiliations:** ^1^CSIRO Agriculture and Food, Canberra, ACT, Australia; ^2^Centre for Tropical Crops and Biocommodities, Queensland University of Technology, Brisbane, QLD, Australia; ^3^Biotechnology Centre, Kenya Agricultural & Livestock Research Organisation, Nairobi, Kenya

**Keywords:** cowpea transformation, *Agrobacterium*, sonication, kanamycin-based selection, geneticin-based selection, cowpeas

## Abstract

An improved cowpea transformation method utilizing *Agrobacterium*-mediated gene delivery to explants derived from the cotyledonary nodes of imbibed cowpea seed is described. The explants were regenerated following a sonication procedure and a stringent selection comprising alternating regimes of kanamycin and geneticin. The method was reproducible and led to the recovery of independent fertile transgenic plants in the greenhouse at a level of about one per cent of starting explants. A transgene encoding an insecticidal protein from *Bacillus thuringiensis* was used to demonstrate the efficacy of the system.

## Introduction

Cowpea is a drought-tolerant grain legume that plays an important role in the diets of many people in Africa and in other parts of the developing world (Murdock et al., 2008). The grain has a protein content of 25% and its leaves and crop residues are used as a vegetable and animal feed, respectively (Nielsen et al., 1997). Although cowpea is important in tropical regions, its low productivity is attributed to biotic and abiotic stresses (Boukar et al., 2016). The majority of cowpea losses are due to insect pests (Murdock et al., 2008). Insect pests have been managed by a variety of approaches including spraying with synthetic and natural products as well as other cultural practices (Ofuya, 1997; Balachandra et al., 2012; Tiroesele et al., 2015). Additionally, breeding for host plant resistance to control thrips, aphids and beetles has been successful, as resistance genes to these exist in the primary gene pool of cowpea (Singh et al., 2002; Hall et al., 2003). However, this is not feasible for a pest such as the *Maruca* pod borer (MPB) as genes conferring resistance to MPB are not present in cowpea although they have been detected in the genome of a wild *Vigna* species (*Vigna vexillata*), which is sexually incompatible with cowpea. In such circumstances modern biotechnology-based approaches, such as genetic engineering, complement the existing methods to control insect pests for cowpea as proposed by Machuka et al. (2000).

Incorporating useful genes into cowpea germplasm using genetic engineering depends on the availability of a reproducible genetic transformation system to generate transgenic plants (Popelka et al., 2006; Aragão and Campos, 2007; Citadin et al., 2013). *In vitro* plant regeneration techniques have been applied to grain legumes via direct shoot organogenesis (Le et al., 2002; Raveendar et al., 2009; Mekala et al., 2016) and indirect organogenesis comprising the establishment of callus cultures, somatic embryogenesis or embryogenic cell suspension cultures (Anand et al., 2000; Ramakrishnan et al., 2005; Aasim et al., 2010). In these studies, different organs or tissues have been used as the starting material including mature seeds (Raveendar et al., 2009; Das et al., 2016), cotyledonary nodal cuttings (Le et al., 2002; Popelka et al., 2006; Chaudhury et al., 2007) mature embryos (Penza et al., 1991; Kaur et al., 2016), plumules (Aasim et al., 2010) and shoot apices or tips (Mao et al., 2006; Mekala et al., 2016). Despite several reports over nearly three decades, the genetic engineering of cowpea is still challenging, consistent with the generally recalcitrant nature of legumes to *in vitro* manipulation (Somers et al., 2003; Bakshi and Sahoo, 2013). This is well illustrated by the many methods that have been trialed using cowpea regeneration via somatic embryogenesis and direct multiple shoot organogenesis (Garcia et al., 1987; Kononowicz et al., 1997; Brar et al., 1999; Popelka et al., 2006; Chaudhury et al., 2007; Raveendar et al., 2009; Behura et al., 2015). Garcia et al. (1987) were among the first to attempt transformation experiments in cowpeas using *Agrobacterium*. Although transgene expression in callus cultures was reported, no regenerated plants were obtained. More recently, *Agrobacterium*-mediated and biolistic transformation have been used successfully to introduce genes conferring traits of potential agronomic importance into cowpea. These traits include insect resistance, herbicide resistance and virus resistance (Popelka et al., 2006; Adesoye et al., 2008; Solleti et al., 2008; Higgins et al., 2012; Citadin et al., 2013; Cruz and Aragao, 2014; Bett et al., 2017). In some of these cowpea transformation protocols, genes encoding neomycin phosphotransferase II (NPT II) coupled with a gene of interest, were used to select cells and tissues that had taken up the transgenes. In these instances, an antibiotic (such as kanamycin or geneticin) resistance gene was used and putatively transformed tissues cultured in selective medium (Garcia et al., 1986; Solleti et al., 2008).

Here, we describe an improved step by step transformation and regeneration system for cowpea. Imbibed mature cowpea seeds were sonicated prior to incubation with *Agrobacterium tumefaciens* carrying a binary T-DNA vector. The explants were then regenerated under a selection regime involving the selectable marker *nptII* combined with alternating antibiotics on plant culture media. The procedures represent a major improvement on the previous protocol (Popelka et al., 2006).

## Materials and Equipment

(1) *A. tumefaciens* strain AGL1 in glycerol stock
(2) Ten sterile 250 mL flasks
(3) Orbital shaker
(4) Mannitol Glutamate Luria (MGL) liquid medium, pH 7 (Table 1)

**Table 1:** Ingredients for the preparation of bacterial MGL medium.

Chemical	Manufacturer	Quantities (per L)
Mannitol	Sigma-Aldrich, St. Louis, MO, United States	5.0 g
L-glutamic acid	Sigma-Aldrich, St. Louis, MO, United States	1.0 g
KH_2_PO_4_	Chem-Supply Pty Ltd., Gillman, SA, Australia	0.25 g
NaCl	Chem-Supply Pty Ltd., Gillman, SA, Australia	0.1 g
MgSO_4_.7H_2_O	Merck, Billencia, MA, United States	0.1 g
Tryptone	Merck, Darmstadt, Germany	5.0 g
Yeast extract	AppliChem, Darmstadt, Germany	2.5 g
Biotin (from 0.1 mg/mL stock)	Sigma-Aldrich, St. Louis, MO, United States	10 μL
(5) Spectinomycin (50 mg/L) (Sigma-Aldrich, St. Louis, MO, United States)
(6) Floor centrifuge
(7) Cowpea seed (IT86D-1010)
(8) Cowpea co-cultivation medium (CCM), pH 5.4 (Tables 2, 3)

**Table 2:** Components of Murashige and Skoog (1962) Macro stock solutions.

Chemical	Manufacturer	Quantities (per L)
20 × MS Macro		
NH_4_NO_3_	Merck, Darmstadt, Germany	33 g
CaCl_2_.2H_2_O	Chem-Supply Pty Ltd., Gillman, SA, Australia	8.8 g
KNO_3_	Sigma-Aldrich, St. Louis, MO, United States	38 g
MgSO_4_.7H_2_O	Merck, Darmstadt, Germany	7.4 g
KH_2_PO_4_	Chem-Supply Pty Ltd., Gillman, SA, Australia	3.4 g
200 × MS Micro		
H_3_BO_3_	Chem-Supply Pty Ltd., Gillman, SA, Australia	1.245 g
MgSO_4_	Merck, Darmstadt, Germany	4.46 g
ZnSO_4_.7H_2_O	AnalaR, NORMAPUR, VWR, Leuven, Belgium	1.72 g
KI	Sigma-Aldrich, St. Louis, MO, United States	166 mg
NaMoO_4_ (1 mg/mL)	AnalaR, BDH Chemicals, VIC, Australia	50 mL
CuSO_4_ (1 mg/mL)	Sigma-Aldrich, St. Louis, MO, United States	5 mL
CoCl_2_ (1 mg/mL)	May & Baker Ltd., Dagenham, England	5 mL
200 × MS Iron		
60% FeCl_3_ solution	AnalaR, BDH Chemicals, VIC, Australia	5.4 mL
200 × MS Na_2_EDTA		
Na_2_EDTA	Chem-Supply Pty Ltd., Gillman, SA, Australia	6.71 g
100 × MS Vitamins		
Thiamine HCl	Sigma-Aldrich, St. Louis, MO, United States	10 mg
Nicotinic acid	AnalaR, BDH Lab Chemicals, England	50 mg
Pyridoxine HCl	Sigma-Aldrich, St. Louis, MO, United States	50 mg
Glycine	Sigma-Aldrich, St. Louis, MO, United States	200 mg

**Table 3:** Composition of cowpea co-cultivation medium (CCM), shoot induction medium (SIM) and shoot elongation and rooting medium (SEM).

Chemical	Manufacturer	Quantities (per L)		
		CCM	SIM	SEM
200 × MS Iron	BDH Chemicals, VIC, Australia	0.5 mL	5 mL	5 mL
200 × MS EDTA		0.5 mL	5 mL	5 mL
200 × MS Micro		0.5 mL	5 mL	5 mL
20 × MS Macro		5 mL	50 mL	50 mL
100 × MS Vitamins		10 mL	10 mL	10 mL
Sucrose	Chem-Supply Pty Ltd., Gillman, SA, Australia	30 g	30 g	30 g
Myo-inositol	Sigma-Aldrich, St. Louis, MO, United States	0.1 g	0.1 g	0.1 g
MES Hydrate	Sigma-Aldrich, St. Louis, MO, United States	3.9 g	0.59 g	0.59 g
Granulated agar (Difco^TM^)	Difco^TM^ Lawrence, KS, United States	8 g	8 g	8 g
6-Benzyl-aminopurine (BAP)	Sigma-Aldrich, St. Louis, MO, United States	1.7 mL (1 mg/mL stock)	1.67 mg	–
Acetosyringone (3.9 mg/mL stock)	Sigma-Aldrich, St. Louis, MO, United States	1mL	–	–
Sodium thiosulfate (1 mM)	AnalaR, BDH Chemicals, Poole, England	1 mL	–	–
Gibberellic Acid (GA_3_)	Sigma-Aldrich, St. Louis, MO, United States	0.125 mL (2 mg/mL stock)	–	0.5 mg
Asparagine	Sigma-Aldrich, St. Louis, MO, United States	–	–	50 mg
Indole-3-acetic acid (IAA)	Sigma-Aldrich, St. Louis, MO, United States	–	–	0.1 mg
(9) Weighing balance
(10) Absolute ethanol
(11) Commercial bleach
(12) Reverse-osmosis (RO) purified water
(13) Petri plates
(14) Aluminum foil
(15) Laminar flow chamber/hood, with sterilizing unit and beads
(16) Growth room chamber with controlled temperatures of 24°C and controlled cool lighting (16 h photoperiod) of 1000–1200 lux provided by white fluorescent bulbs.
(17) Scalpel blades, spatula and scalpel holder
(18) Sonicator (Bransonic Ultrasonic Cleaner, Model 1510E-DTH; output 70 W)
(19) Medical tape
(20) Permanent marker (for labeling cultures)
(21) Personal protective equipment (Latex hand gloves; face masks, safety goggles and labcoat)
(22) Measuring cylinders (500-, 1000- and 2000 mL) (Pyrex)
(23) Cylindrical glass beakers (500-, 1000- and 2000 mL) (Pyrex)
(24) Pipettes and pipette tips (10-, 50-, 100-, 500- and 1000 μL)
(25) Shoot induction medium (SIM), pH 5.6 (Table 3)
(26) Shoot elongation and rooting medium (SERM), pH 5.6 (Table 3)
(27) Kanamycin (Sigma-Aldrich, St. Louis, MO, United States)
(28) Spectinomycin (Sigma-Aldrich, St. Louis, MO, United States)
(29) Merrem (Ranbaxy Australia Pty, Sydney, NSW, Australia)
(30) Geneticin (G-418) (Sigma-Aldrich, St. Louis, MO, United States)
(31) Light sandy soil mix (25% perlite, 25% vermiculite, 30% coarse sand and 20% commercial potting mix (Bunnings, Canberra, ACT, Australia)
(32) Soil (70% commercial potting mix, 10% perlite, 10% river sand, 10% peat, 1.5 g/L Osmocote, 0.5 g/L Nitram and 3 g/L Carb lime)
(33) Pots (7 cm diameter × 9 cm height and 20 cm diameter × 20 cm height)
(34) Nucleic acid isolation kit (Puregene, MN, United States)
(35) Phusion^®^ Hot Start Flex 2X master mix (Cat # M0536S, New England Biolabs)
(36) *Vip3Ba* forward and reverse primers: 5′GAACGCTCAGCTCAACTCCA3′ and 5′GGTGGAGTTGATGAGCACGT3′
(37) *nptII* forward and reverse primers: 5′GCTTGGGTGGAGAGGCTATT3′ and 5′TCATTTCGAACCCCAGAGTC3′
(38) PCR thermocycler (BioRad)
(39) SDS Gels (Thermo Fisher Scientific)
(40) Gel tanks and appropriate combs
(41) Gel electrophoresis equipment
(42) Agarose gel
(43) Trans-illuminator gel documentation system (GelDoc 2000 software, BioRad)
(44) Pestle and mortar
(45) Protein extraction buffer (0.1 M *N*-[Tris(hydroxymethyl) methyl]-2-aminoethanesulfonic acid (TES) (AnalaR, NORMAPUR, VWR, Leuven, Belgium) pH 7.6, 0.2 M NaCl, 1 mM phenylmethanesulfonyl fluoride (PMSF) (Sigma-Aldrich, St. Louis, MO, United States), 1 mM EDTA) in a 1.5 mL Eppendorf tube
(46) *Escherichia coli*-expressed Vip3Ba protein
(47) Vip3Ba-specific monoclonal antibody (AbMart Inc., Shanghai, China)

## Stepwise Procedures (Based on Bett, 2016)

### Preparation of *Agrobacterium* Cultures

(1) An aliquot (400 μL) of a glycerol stock of the *A. tumefaciens* strain AGL1 (Lazo et al., 1991) containing a *Vip3Ba* gene construct (Bett et al., 2017) was added to 100 mL of MGL liquid medium, pH 7 (Table 1), in a sterile 250 mL flask, in the laminar hood.
(2) Spectinomycin (to 50 mg/L) was added to the *Agrobacterium* culture as the selective agent.
(3) Aluminum foil was used as a lid to cover the flasks containing the cultures.
(4) The culture was allowed to grow overnight in an orbital shaker at 28°C at 200 rpm and then centrifuged for 15 min at 7500 *g* at room temperature.
(5) The pellet was re-suspended in 100 mL of cowpea co-cultivation medium (CCM) (Tables 2, 3), pH 5.4 using an orbital shaker at 200 rpm for a minimum of 1 h at 28°C.
(6) Prior to inoculation of explants, an additional 100 mL of CCM was added to the *Agrobacterium* suspension to make a total of 200 mL.

### Preparation of Cowpea Explants for *Agrobacterium*-Mediated Transformation

The transformation method was a modification of the protocol described by Popelka et al. (2006) (Table 4). The sterilization procedures commenced 18 h prior to the co-cultivation step, to give sufficient time for the seeds to imbibe and soften for explant preparation.

**Table 4:** Modifications of the transformation system used for cowpea.

	Transformation protocol according to Popelka et al. (2006)	Modifications made in the current study
Seed sterilization	Dry seed sterilized for 45 min in 50% bleach.	Dry seed sterilized in 70% ethanol for 1 min, followed by 20% commercial bleach for 30 min.
*Agrobacterium*-inoculation stage	Wounded the explants using a scalpel blade.	Explants sonicated for 30 s while submerged in *Agrobacterium* suspension.
Co-cultivation stage	Infected explants co-cultured for 6 days. Incorporated 1 g/L of L-cysteine in the co-cultivation media.	Infected explants co-cultured for 3 days. No L-cysteine in co-cultivation media.
Shoot induction stage	Incorporated 250 mg/L sodium thiosulfate in the shoot induction medium.	No sodium thiosulfate in the shoot induction medium (SIM).
Selection conditions	Explants on shoot induction medium without selection for 12 days. The explants were transferred to selection media (phosphinothricin at a constant level) for the next eight tissue culture transfers including the rooting stage.	Explants were immediately transferred to selection (kanamycin at 100 mg/L) on shoot induction medium for 12 to 14 days. The explants were then transferred to a higher level of selection (150 mg/L kanamycin) for two tissue culture transfers (28 days). The selection agent was then changed to geneticin at 30 mg/L for the remaining transfers (including rooting) for a minimum of 84 days.

**FIGURE 1:**
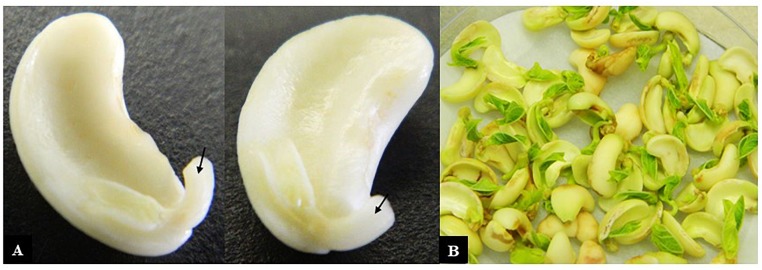
Cowpea explants used for transformation. **(A)** Cotyledons with attached axes that had their radicle tips removed (arrows) ready for incubation with *Agrobacterium* and **(B)** after co-cultivation with *Agrobacterium* for 3 days.
(10) Approximately 50 explants were placed in sterile 250 ml flasks containing 10 mL of CCM, to keep the explants hydrated until all 400 explants were prepared.
(11) To transform the explants, CCM was replaced with approximately 25 mL of the *Agrobacterium* suspension to submerge 50 explants.
(12) The explants were sonicated for 30 s using a Bransonic Ultrasonic Cleaner, Model 1510E-DTH; output 70 W.
(13) The sonicated explants were incubated for 1 h on a rotary shaker at 28°C and 200 rpm.
(14) The explants were placed onto sterile filter papers to blot the excess medium.
(15) Co-cultivation plates were prepared by pouring 30 mL of CCM media solidified with 8 g/L granulated agar and placing two new sterile filter papers on the media.
(16) 30 to 40 explants were transferred onto each filter paper plates and co-cultivated for 3 days at 28°C with a 16 h photoperiod.

(1) Dry cowpea seed was weighed (30 g) into a 250 mL Schott bottle.
(2) 50 mL of 70% ethanol was added for 1 min.
(3) The mixture was shaken vigorously for 30 s.
(4) The ethanol was poured off, replaced with 50 mL of 20% commercial bleach and incubated for 30 min at room temperature.
(5) The seeds were rinsed 5 times in sterile RO-purified water.
(6) Seeds were allowed to imbibe in 50 mL of sterile RO water overnight.
(7) Imbibed seeds were drained, and seed coats aseptically removed.
(8) Each seed was split in two by separating the cotyledons.
(9) Using the cotyledon with the attached embryonic axis (hereafter referred to as the explant), the lower 2/3 of the radicle was excised (Figure 1A).

### Regeneration and Maintenance of Cowpea Cultures

(1) The explants were placed with the growing shoot facing down onto SIM, pH 5.6 (Table 3) containing 100 mg/L kanamycin for selection and 25 mg/L Merrem to prevent the growth of *Agrobacterium*.
(2) Explants were incubated in a growth chamber at 28°C with a 16 h photoperiod for 12 to 14 days.
(3) At the next transfer (day 17), the cotyledon, primary shoots and any regrown radicle and was removed and transferred the remaining portion of the explants to SIM with increased kanamycin (150 mg/L) and Merrem (25 mg/L).
(4) Explants were incubated in a growth chamber at 28°C with a 16 h photoperiod for 12 to 14 days.
(5) At the third transfer (day 29), any brown callus was removed. The remaining tissue supporting multiple small shoots was transferred to SIM with kanamycin (150 mg/L) and Merrem (25 mg/L).
(6) Explants were incubated in a growth chamber at 28°C with a 16 h photoperiod for 12 to 14 days.
(7) At the fourth transfer (day 45), multiple shoots were transferred to SIM with Merrem (25 mg/L) and more stringent selection using geneticin (30 mg/L).
(8) Explants were incubated in a growth chamber at 28°C with a 16 h photoperiod for 12 to 14 days.
(9) Two further transfers (each of 12–14 days) were made onto SIM with 30 mg/L geneticin. Large clumps consisting of many shoots were separated into smaller clumps or selected as single shoots depending on their vigor. Dead shoots were removed and discarded in these transfers.
(10) In the seventh transfer, multiple shoots were separated and incubated on SIM with 30 mg/L geneticin at 28°C with a 16 h photoperiod for 12 to 14 days.
(11) For the eighth transfer, single shoots were placed onto shoot elongation and rooting medium (SEM), pH 5.6 (Table 3) with geneticin (30 mg/L) and 25 mg/L Merrem for 14 days to allow elongation and rooting. Depending on their height, shoots were placed on either Petri plates or in 250 mL containers with lids and incubated in a growth chamber at 28°C with a 16 h photoperiod.
(12) Shoots developing healthy roots were selected, and solid SEM rinsed off their roots with lukewarm water. These were transferred into small pots (7 cm diameter × 9 cm height) containing a light sandy soil mix. Subsequently, these were maintained in the culture room for up to 4 weeks for acclimatization after which they were transferred to the glasshouse in larger pots (20 cm diameter × 20 cm height) containing soil.

### Detection of *vip3Ba* and *nptII* Genes in the Transgenic Plants

(1) Genomic DNA was extracted from putatively transgenic cowpea plants using a nucleic acid isolation kit (Puregene, MN, 55447, United States).
(2) PCR mixes were prepared using 5 μL of Phusion^®^ Hot Start Flex 2X master mix (Cat # M0536S, New England Biolabs), forward and reverse Vip3Ba or nptII primers (2 μL each) and 10 μL dH_2_O.
(3) Approximately 100 ng genomic DNA was added, and samples subjected to the following PCR cycling regime: 98°C for 30 s, 98°C for 10 s, 60°C for 20 s and 72°C for 90 s (30 cycles), followed by 72°C for 10 min and 25°C for 1 min.
(4) Amplicons were analyzed by electrophoresis through a 1.8% agarose gel at 60 mA and photographed using a trans-illuminator gel documentation system.

### Detection and Estimation of Vip3Ba Expression in T_1_ and T_2_ Transgenic Plants

(1) T_1_ seeds were harvested from the T_0_ transgenic lines to obtain T_1_ and T_2_ plants for protein isolation.
(2) Non-transgenic line (IT86D-1010) was propagated as a negative control.
(3) Young fully expanded unifoliate leaves (7-days old) were harvested from the transgenic and non-transgenic lines.
(4) Total soluble protein (TSP) was extracted by macerating 80–110 mg of cowpea leaf (from individual plants) in 500 μL of protein extraction buffer.
(5) A 40 μg aliquot of TSP was subjected to SDS-PAGE, together with known levels (1, 3, 10, and 30 ng) of *E. coli*-expressed Vip3Ba protein.
(6) The SDS-PAGE gel was blotted to nitrocellulose membranes and assessed for the presence of Vip3Ba by western blot using a Vip3Ba-specific monoclonal antibody.
(7) The amount of Vip3Ba protein was estimated by visual comparison with the known concentrations of *E. coli*-expressed Vip3Ba protein previously included on the gels.
(8) Data was expressed as ng Vip3Ba per mg of TSP.

### Potential Experimental Pitfalls or Artifacts

The following unforeseen circumstances may occur while carrying out transformation and regeneration experiments:

(i) Contamination of growth media on Petri plates and explant contamination. Apply sterilization techniques during media dispensation and explant sterilization procedures to overcome these. In addition, hormones and antibiotics should be filter-sterilized and added to the plant culture media after autoclaving and cooling to 50°C.
(ii) The *Agrobacterium* culture may not grow to an optimum Optical Density (OD) prior to inoculation of explants. To avoid this, inoculate the MGL medium with *Agrobacterium* no less than 24 h prior to the co-cultivation step and grow overnight at 28°C.
(iii) Cowpea seeds may be too hard to prepare explants, therefore commence sterilization procedures 18 h prior the co-cultivation step. This will give sufficient time for the seeds to imbibe for explant preparation.
(iv) The explants may dry up during preparation. Keep the explants hydrated in co-cultivation medium prior to the Agro-inoculation procedure.

## Results and Discussion

### Transformation and Regeneration

**FIGURE 2:**
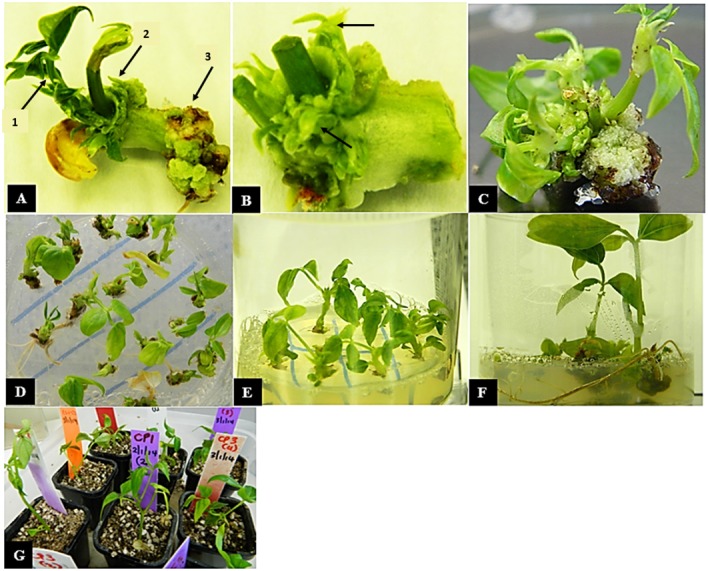
*In vitro* regeneration of cowpea explants following co-cultivation with *Agrobacterium*. **(A)** Cowpea explant with cotyledon and primary shoots (arrow 1) on shoot induction medium (SIM) with selection at 2 weeks after co-cultivation, regenerated small buds (arrow 2) and callus (arrow 3) are visible **(B)** explant with cotyledon and primary shoot removed at 4 weeks leaving a clump with shoot buds (arrows) **(C)** multiple shoots formed on callus **(D)** multiple shoots separated onto SIM with 30 mg/L geneticin at 8 weeks **(E)** individual shoots grown with 30 mg/L geneticin at 10 weeks, **(F)** individual shoots rooting on elongation and rooting medium at 14 weeks and **(G)** rooted plantlets in soil at 16 weeks.

The transformation procedures described above were repeated fourteen times. A total of 6696 explants were co-cultivated with *Agrobacterium* containing the *vip3Ba* transgene. After the 3-day co-cultivation period, primary shoots were observed developing from the embryonic axis (Figure 1B). The explants were then transferred to SIM with kanamycin for a maximum of 14 days. By this time, primary shoots had elongated (Figure 2A) and potentially transformed small shoot buds appeared at the cotyledonary node (Figure 2A), and callus had formed at the radicle end (Figure 2A). The cotyledons, primary shoots and radicle-associated callus were removed leaving a clump with the small shoot buds (Figure 2B) on a callus base. The remaining part of the explants was transferred to SIM with kanamycin for two transfers (on Days 17 and 29). All subsequent transfers were onto medium containing geneticin. The clumps of multiple shoot buds (Figure 2C) were divided and any dead tissue and surplus callus was removed (Figure 2D). By transfer 6 (Day 70), single shoots were separated onto fresh SEM (Figure 2E). Roots developed during two cycles on shoot elongation media (Figure 2F). The rooted plantlets were acclimatized in a tissue culture room prior to transfer to the glasshouse (Figure 2G). Following selection, 77 independent putative transgenic lines were obtained. All plants that regenerated from one explant were considered to be clones (siblings) belonging to one transgenic event. Using this transformation protocol, whole plantlets (approximately 7 to 10 cm in height) belonging to independent transgenic lines were obtained in approximately 4 months.

The successful transformation of many plant species, including cowpea, is dependent on the genotype, the type of explant used, the antibiotic or herbicide selection regime and plant growth media composition (Manman et al., 2013). In this study, significant modifications were made to the previous cowpea transformation protocol (Popelka et al., 2006) to improve its efficiency. These technical changes resulted in a cowpea transformation system with an efficiency of just over 1%, with 73 independent transgenic lines obtained. This rate is similar to, or better than some other cowpea transformation studies in which efficiencies ranged from 0.15 to 3.9% (Popelka et al., 2006; Chaudhury et al., 2007; Ivo et al., 2008; Solleti et al., 2008; Adesoye et al., 2010; Raveendar and Ignacimuthu, 2010; Behura et al., 2015). The sonication of legume cotyledonary nodes has been shown to increase the transformation efficiency. Using sonication-assisted *Agrobacterium*-mediated transformation in soybean, Trick and Finer (1998) generated hygromycin-resistant clones and reported enhanced transformation compared to very low transient expression of GUS gene when sonication procedures were not employed. Further, a 100% increase in GUS expression frequency was also achieved following sonication treatment from 5 to 25 s in the transformation of winter cherry (Sivanandhan et al., 2015). In the current study the co-cultivated cowpea explants were sonicated and also subjected to 100 mg/L kanamycin for early stage selection, and then kanamycin at 150 mg/L and geneticin at 30 mg/L for the remaining transfers thereby obtaining transgenic plants in soil within approximately 4 months.

**Table 5:** Summary of the molecular analysis of transgenic cowpea lines.

No. of primary transgenics	No. of lines PCR positive for *nptII*	No. of lines PCR positive for *vip3Ba*	No. of lines selected for testing by western blot	No. of lines with detectable levels of Vip3Ba protein
77	73	73	42	9

### Characterization of Transgenic Lines by PCR

**FIGURE 3:**
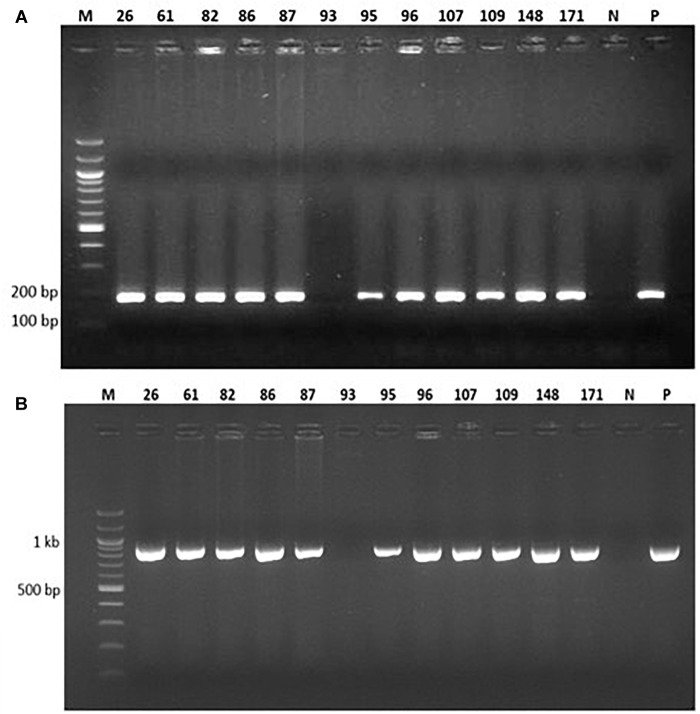
PCR analysis of 12 representative putatively transgenic cowpea lines using **(A)** *vip3Ba*-specific primers designed to amplify a 187 bp product and **(B)** *nptII*-specific primers designed to amplify a 970 bp product. Lane M: DNA Molecular weight marker; Lane N: No template (negative control); Lane P: Plasmid DNA containing both *vip3Ba* and *nptII* genes (positive control); numbers above lanes represent the line number of randomly selected, independent transgenic cowpea plants.

Genomic DNA was extracted from the 77 putative transgenic cowpeas and used as a template in PCR with primers designed to amplify a 187 bp fragment of the *vip3Ba* gene and a 970 bp region of the *nptII* gene (Figure 3). Of the 77 plants, 73 were positive for both the *vip3Ba* and *nptII* genes (Table 5), resulting in an average transformation frequency of 1.1%. All 73 T_0_ plants were fertile and seed from 42 lines was selected for further analysis.

### Expression of Vip3Ba in T_1_ and T_2_ Generations

**FIGURE 4:**
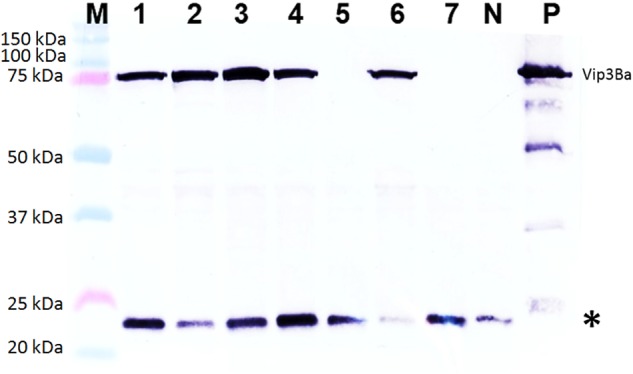
Western blot analysis of Vip3Ba expression in seven T_1_ progeny derived from transgenic cowpea line 43. Lane M: Protein precision markers; Lanes 1 to 7: Protein (40 μg) extracts from leaves of seven T_1_ plants of line V43; Lane N: protein from line IT86D-1010 (negative control); Lane P: 100 ng of Vip3Ba protein from *Escherichia coli* expressing Vip3Ba (positive control). Vip3Ba band migrates at ∼75 kDa. The 22 kDa band (^∗^) in all lanes including the negative control represents an unknown protein that cross-reacts with the monoclonal antibody.

Of 42 independent T_0_ lines analyzed by western blotting, a band of the expected size for Vip3Ba (∼75 kDa) was detected in extracts from 9 lines (Table 5) namely V9, V24, V25, V43, V56, V87, V107, V176 and V191. Based on comparison to the standards included in the gels, the amount of Vip3Ba present in seven (V9, V24, V25, V43, V56, V87, V107) of these lines ranged between 0.25 and 5.0 μg/mg TSP (Bett et al., 2017). A representative blot of total soluble leaf protein extracts from seven T_1_ progeny derived from one line (V43) is presented in Figure 4. Subsequently, four lines (V24-9, V25-8, V43-3, V87-2) of the T_1_ progeny produced T_2_ seed and further produced T_2_ plants. Following a Western blot analysis of at least five T_2_ plants from each of the four T_1_ lines, a band of the expected size for Vip3Ba (∼75 kDa) was detected in extracts from these progenies indicating expression in the segregating progeny of the four lines (data not shown).

There were varying levels of Vip3Ba protein amongst the independent transgenic cowpea lines (data not shown). Variable transgene expression is a common phenomenon in genetically modified plants and levels can vary widely between lines (Matzke and Matzke, 1998; Schuler et al., 1998). This variation is generally considered to be the result of (i) the position effect i.e., where in the genome the transgene cassette has integrated, and (ii) complex integration events which can trigger post-transcriptional gene silencing (PTGS) and/or transcriptional gene silencing (TGS) (Stam et al., 1997; Matzke and Matzke, 1998; Schuler et al., 1998). The latter of these phenomena are of particular importance as both PTGS and TGS can result in the complete silencing of transgene expression.

The levels of Vip3Ba measured in this study were within the range of those observed in cowpea lines expressing Cry 1Ab, but higher than those reported in chickpea expressing Cry 1Ac (Kar et al., 1997; Sanyal et al., 2005; Indurker et al., 2010; Mehrotra et al., 2011; Ganguly et al., 2014).

This improved protocol for cowpea transformation yielded a higher transformation efficiency than that described in Popelka et al. (2006), Chaudhury et al. (2007) and Ivo et al. (2008). Chaudhury et al. (2007) employed kanamycin (85 mg/L) for shoot regeneration and subsequently obtained shoots rooted on media containing kanamycin at a reduced concentration of 10 mg/L, resulting in inheritance of transgenes to progeny in Mendelian fashion at a rate of 0.76%. The system presented here is a modification of existing protocols (Popelka et al., 2006; Citadin et al., 2011), which included using a growth medium in the absence of certain reducing agents (L-cysteine and sodium thiosulfate at co-cultivation and shoot induction stages, respectively), a different antibiotic selection regime, and employing sonication to permeate the plant cell wall thereby facilitating *Agrobacterium*-mediated T-DNA transfer into the host plant cell. Higgins et al. (2012) employed a kanamycin/geneticin regime at 150 and 25–50 mg/L, respectively, for shoot initiation in cowpea. The surviving green shoots were further subjected to 50 mg/L geneticin or 150 mg/L kanamycin for shoot elongation and 1–3 transgenic plants per 1000 explants were obtained, which is equivalent to 0.1–0.3% transformation efficiency (Higgins et al., 2012). In contrast, Bakshi et al. (2011) used sonication and vacuum infiltration assisted *Agrobacterium*-mediated transformation for cowpea, which substantially increased the transformation efficiency.

With this protocol, rooted plants were recovered within 4 months of explant preparation. This duration is within the range of other researchers, who reported varied timelines in recovery of rooted plants between 1 and 8 months (Chaudhury et al., 2007; Raveendar and Ignacimuthu, 2010; Bakshi et al., 2011; Higgins et al., 2012).

The improved result in this study is attributed to wounding of the cotyledonary explants by sonication, using alternating antibiotics for selection and the use of growth media without certain reducing agents previously incorporated by Popelka et al. (2006).

## Author Contributions

TH and RH conceived and designed the study. BB and SG performed the transformation experiments. BB and AM performed the molecular characterization experiments. BB, RH, and TH analyzed and interpreted the results. BB, SG, RH, and TH prepared the manuscript. All authors read and approved the manuscript.

## Conflict of Interest Statement

The authors declare that the research was conducted in the absence of any commercial or financial relationships that could be construed as a potential conflict of interest.
